# Corrosion Behavior of the AlCoCrFeNi_2.1_ Eutectic High-Entropy Alloy in Chloride-Containing Sulfuric Acid Solutions at Different Temperatures

**DOI:** 10.3390/ma15144822

**Published:** 2022-07-11

**Authors:** Longfei Song, Wenbin Hu, Xiaowen Zhang, Bokai Liao, Shan Wan, Lei Kang, Xingpeng Guo

**Affiliations:** School of Chemistry and Chemical Engineering, Guangzhou University, Guangzhou 510006, China; wilbur326@163.com (W.H.); zhangxw0222@163.com (X.Z.); bokailiao@gzhu.edu.cn (B.L.); shanwan@gzhu.edu.cn (S.W.); kanglei@gzhu.edu.cn (L.K.)

**Keywords:** eutectic high-entropy alloy, corrosion, passive film

## Abstract

In this work, the influence of temperature on the corrosion behavior of AlCoCrFeN_i2.1_ eutectic high-entropy alloy in a chloride-containing sulfuric acid solution was investigated using electrochemical measurement, X-ray photoelectron spectroscopy, and scanning electron microscopy. Results show that the passive film of AlCoCrFeN_i2.1_ is stable in chloride-containing sulfuric acid solutions at low temperatures, while an unstable film forms on the alloy at high temperatures. Furthermore, temperature changes the proportion of hydroxide and oxide in Fe and Cr, but it has no noticeable effect on Al and Ni, which is a significant factor on the passive behavior. L12 phase exhibits good corrosion resistance at different temperatures. Pitting occurred on B2 phase in the chloride-containing sulfuric acid solution at a low temperature of 5 °C, while pitting and dissolution take place on AlCoCrFeNi_2.1_ in the acid solution at room temperature and above.

## 1. Introduction

The high-entropy alloy (HEA) is a new alloy with more than four main elements. In recent years, HEAs have attracted attention due to their outstanding performance, including superior mechanical properties, abrasion resistance, and corrosion resistance [1,2]. Single-phase body-centered-cubic (BCC) and face-centered-cubic (FCC) HEAs have been the subject of much research over the past decade. Several studies have reported that BCC HEAs possess high strength, while FCC HEAs are highly ductile. According to comprehensive research, it is difficult to achieve both high strength and ductility in single-phase HEAs [3,4,5]. To further improve the properties of HEA, Lu et al. [6,7] proposed the concept of eutectic high-entropy alloy (EHEA) that combined the strength of BCC HEAs and ductility of FCC HEAs. EHEAs have broad application prospects due to their excellent mechanical properties over a wide temperature range [8,9,10]. However, EHEA research mainly focuses on mechanical properties, while there are few studies on the corrosion behavior of EHEAs. Consequently, the development and application of the EHEA have been somewhat restricted.

Passivation behavior and local corrosion are the primary research topics on the corrosion of metals with passivation ability [11,12,13,14]. The influence of the component elements and microstructure on the corrosion and passivation behavior of other HEAs has been investigated in the past. Yet, there are few reports on the corrosion behavior of the AlCoCrFeNi_2.1_ EHEA. The content of Al, Co, Cr, Fe, and Ni in the EHEA all affect its passivation behavior, which is essential to the corrosion resistance of the EHEA. Alloys with more than 12 at.% Cr content can form a passive film in some solutions [15]. Cr is the primary component of passive films [16,17]. Additionally, some research have reported that Ni, Al, Co, and Fe can also be the constituent elements in passive films [16,18,19]. Shi et al. [20] established that an increase in Al content reduced the protective ability of the passive film on the surface of the AlxCoCrFeNi HEA in 3.5% NaCl solutions. Chen et al. [21] discovered that the Cu_0.5_NiAlCoCrFeSi HEA was more prone to pitting than 304 stainless steel in chloride solution. Xu et al. [22] demonstrated that Ni was the central element of the passive film on CoCrFeMnNi HEA in sulfuric acid solution. In addition, Chai et al. [23] revealed that the FeCoNiCr_0.5_ HEA possessed good local corrosion resistance, while the dissolution of the Cr-rich zone in the dendrites was accelerated. Lin et al. [24] asserted that the Al-Ni-rich phase on FCC was the preferential corrosion area in chloride-containing sulfuric acid solutions. Additionally, unique microstructures alter the corrosion behavior of the AlCoCrFeN_i2.1_ EHEAs, compared to other HEAs. The eutectic structure may lead to the galvanic effect, which making the EHEA face local corrosion risks.

Corrosion is a serious threat to the security of metals, and is a vital issue in the application of new materials. Scholars have conducted much research about the corrosion behavior of HEAs, but studies on EHEA corrosion are relatively rare. It has been reported that HEAs exhibit different corrosion resistance in various corrosive environments. Wang et al. [25] discovered that the CoCrFeNiMo_0.01_ EHA displayed pseudo-passive behavior in an H2S-containing environment. Luo et al. [26] found that the pitting corrosion resistance of the interstitial equiatomic CoCrFeMnNi HEA was influenced by carbon content in a Cl--containing solution. Most studies have focused on the corrosion behavior of HEAs at room temperature (~25 °C). However, HEAs would are often used in corrosive mediums at different temperatures. Until now, only a few studies have paid attention to the influence of temperature on the corrosion of HEAs in aqueous solutions. For instance, Chou et al. [27] showed that the pitting potential of the Co_1.5_CrFeNi_1.5_Ti_0.5_Mo_0.1_ HEA changed linearly with the logarithm of chloride concentration at 70 °C and 80 °C. Moreover, studies on the corrosion behavior of HEAs are usually based on stainless steel. The relationship between corrosion behavior and environmental temperature is an essential topic in the study of stainless steel. Hou et al. [28] considered that the passive film resistance of 316L SS in methyl diethanolamine solution dropped as the solution temperature rose. Cui et al. [29] established that temperature caused a decrease in the protection of passive properties in 2507 super duplex stainless steel by altering the doping concentrations and structure. Lei et al. [30] reported that pitting corrosion on S44660 stainless steel was more likely to occur in bromide solution than in chloride solution at low temperature. The corrosion behavior of EHEA is greatly affected by temperature, as with stainless steel. Therefore, investigating the influence of temperature on the corrosion behavior of the AlCoCrFeNi_2.1_ EHEA has great significance to the study of the EHEA corrosion mechanism and the choice of its usage scenarios.

In this paper, the corrosion behavior of the AlCoCrFeNi_2.1_ EHEA in chloride-containing sulfuric acid solution at different temperatures were studied using field emission scanning electron microscopy (FESEM), energy dispersive spectrometry (EDS), electron back-scattering diffraction (EBSD), atomic force microscopy (AFM), electrochemical methods, and X-ray photoelectron spectroscopy (XPS). The effects of temperature on the passive and local corrosion mechanism of EHEA were discussed. This work accumulates basic data for the study of corrosion resistance of EHEA and provides for application of AlCoCrFeNi_2.1_ EHEA in corrosive environment.

## 2. Materials and Methods AlCoCrFeNi_2.1_

### 2.1. Materials and Solution

**Figure 1:**
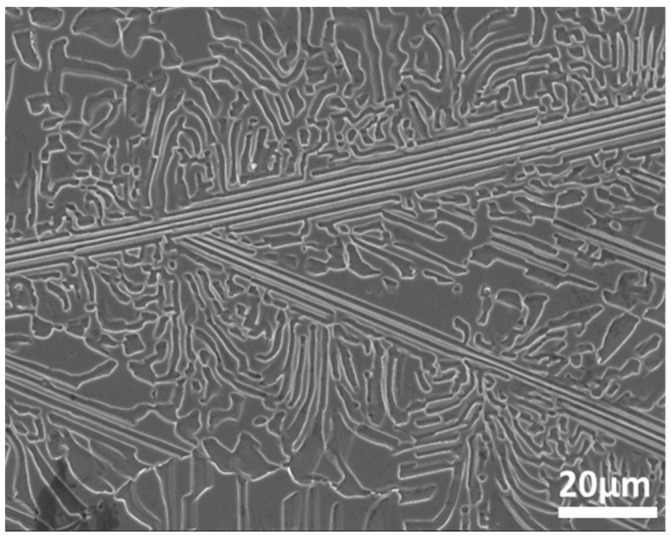
Microstructure of AlCoCrFeNi_2.1_.

AlCoCrFeNi_2.1_ EHEAs were prepared from high-purity metal (99.95%, Al, Co, Cr, Fe, Ni) in a vacuum melting furnace. The EHEAs were re-melted 5 times to ensure homogeneity. The microstructure and chemical composition of the EHEA were analyzed by FESEM (JSM-7001F, JEOL, Tokyo, Japan) and EDS after polishing and etching with aqua regia for 20 s. Figure 1 displays the microstructure of the EHEA. The alloy consists of a lamella area and irregular area, which is consistent with previous literatures [31,32]. Research has reported that the lamellar region of the AlCoCrFeNi_2.1_ EHEA contained L12 and B2 phase, respectively [33,34]. The crystallographic information of the EHEA was further analyzed by EBSD, and the sample for EBSD was prepared by electrochemical polishing in an electrolyte with perchloric acid and ethanol (1:4). Scanning Kelvion probe force microscopy (SKPFM) enabled the characterizations of the potential differences between various phases. The SKPFM test of the EHEA was examined by atomic force microscopy (AFM) (Bruker Multimode VIII, Bruker, Billerica, MA, USA). The sample for SKPFM, which had dimensions of 10 mm × 10 mm × 2 mm, was ground with SiC paper to 5000 grit, mechanically polished with 2.5 μm and 1.5 μm diamond paste, and then polished with oxide polishing suspension (OPS) solution. The distance between the probe tip and the EHEA surface was 100 nm. The nominal resonance frequency was 75 kHz, and the spring constant was 3 N/m. The corrosion behaviors of the EHEAs were studied in a 0.005 M H_2_SO4 + 0.05 M NaCl solution at several temperatures (5 °C, 25 °C, 40 °C and 60 °C).

### 2.2. Electrochemical Tests

The samples for the electrochemical testing methods were embedded in epoxy resin, leaving an exposed area of 1 cm^2^. The working surface was ground with emery paper. In this study, all of the electrochemical tests were carried out on a CHI660 electrochemical workstation (Wuhan, China) using the three-electrode test system. The working electrode, reference electrode, and platinum plate were EHEA, a KCl saturated calomel electrode (SCE), and a counter electrode, respectively. Before all the electrochemical tests, the working electrodes were maintained for 1 h to reach a stable value of the open circuit potential. Potentiodynamic polarization (PDP) curves were measured from −0.3 to 0.6 VvsOCP with a scanning rate of 0.5 mV/s. Electrochemical impedance spectroscopy (EIS) (JSM-7001F, JEOL, Tokyo, Japan) measurements were carried out to study the surface state of the EHEA. During the EIS tests, the testing frequency ranged from 0.01 Hz to 10,000 Hz, and the perturbation amplitude was 10 mV. The EIS data were fitted by ZsimpWin software, (ZSimpWin 3.50 Ann Arbor, MI, USA). Potentiostatic measurements were performed to study the passivation behavior of the EHEAs at different temperatures, and the potential was chosen based on the PDP curve. Moot-Schottky curves were tested from 800 to −600 mV_vsSCE_ with 0.5 V/step at a frequency of 1000 Hz. A fast scan rate was selected to avert a change in the surface state during the tests, which is in accordance with the “frozen-in defect structure”. The electrochemical tests repeated three times to ensure accuracy.

### 2.3. XPS Tests

To study the passivation behaviors of the EHEAs in chloride-containing sulfuric acid solution at various temperatures, the composition of the film on the EHEA samples after potentiostatic polarization for 4 h at 5 °C and 60 °C was tested by using XPS (AXIS NOVA, SHIMADZU, Kyoto, Japan) equipped with Al Kα radiation working at 15 kV and 25 W. The standard peak (C1s, 284.8 eV) was used to correct all the XPS peaks, and the XPS data were analyzed by XPSpeak 4.1 software (freeware written by Raymond Kwok, Hong Kong, China). The standard peak (C1s, 284.8 eV) was used to correct all the XPS peaks, and the XPS data were analyzed by XPSpeak 4.1 software.

### 2.4. Corrosion Observation

The surface morphologies of the EHEA after potentiostatic polarization at 0.3 V_vsSCE_ for 20 min were observed using FESEM (JSM-7001F JEOL, Tokyo, Japan), and the element distribution on the corrosion surface was measured by EDS.

## 3. Results and Discussion

### 3.1. Microstructure

**Figure 2:**
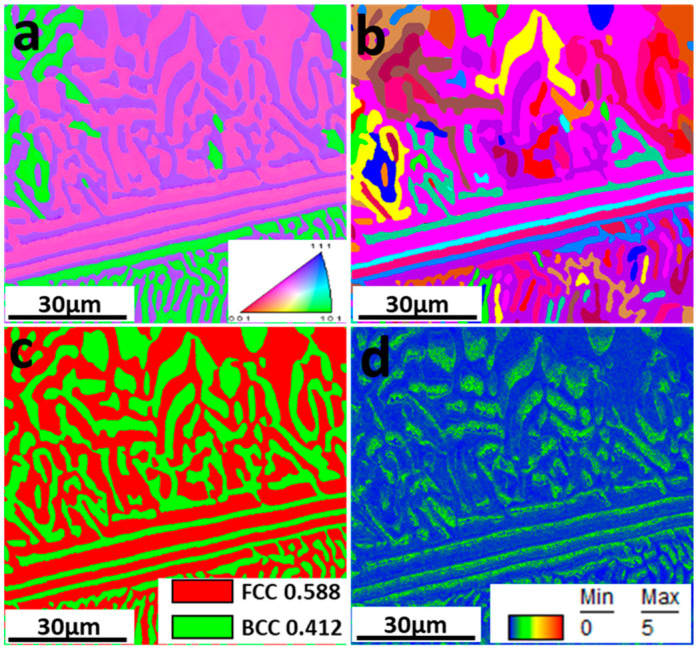
EBSD results of AlCoCrFeNi_2.1_: (**a**) IPF map; (**b**) Grain map; (**c**) Phase map; (**d**) KAM map.

**Figure 3:**
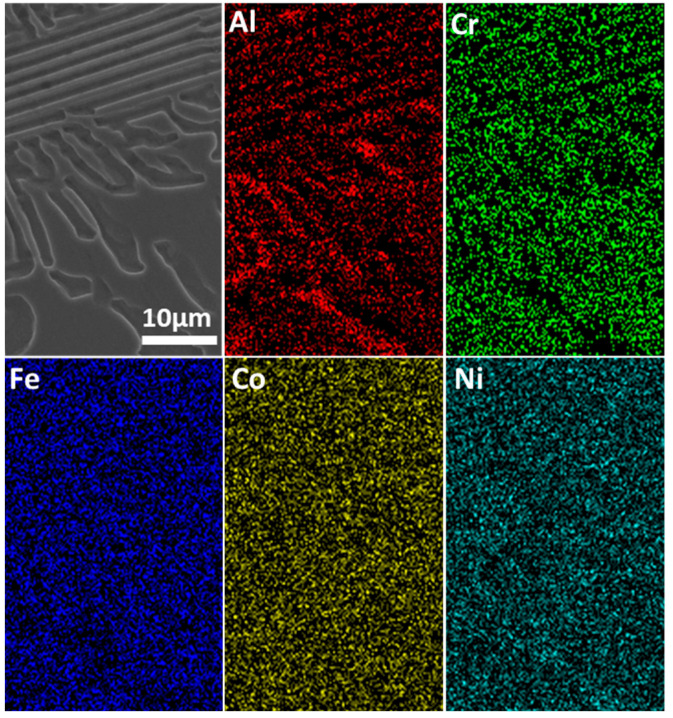
EDS of AlCoCrFeNi_2.1_.

**Figure 4:**
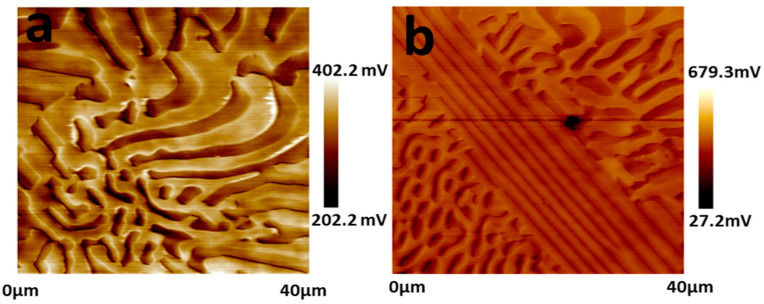
Volta potential mappings of AlCoCrFeNi_2.1_ tested by SKPFM. (**a**,**b**) potential map of the irregular region and the lamella region.

Electron back-scattering diffraction was used to study the relationship between microstructure and corrosion behavior. Figure 2 shows the EBSD data, including inverse pole figure (IPF), grain, phase, and kernel average misorientation (KAM) map. The EHEA presents three crystal orientations, as Figure 2a illustrates. The volume fractions of FCC and BCC phases were calculated by professional software in the phase map. The volume fraction of the L12 (FCC) phase and the B2 (BCC) phase are 41.2% and 58.8%, respectively. The B2 (BCC) phases (green color) are paralleled in the lamellae area, as shown in Figure 2a. According to the KAM map, the B2 phase possesses a higher KAM value than the L12 phase, indicating that the B2 phase presents a higher dislocation density and, therefore, higher electrochemical activity. The EDS map in Figure 3 shows Al and Ni are enriched in the B2 phase, while FCC is enriched with Cr. SKPFM was used to test the potential of the L12 and B2 phases. Figure 4a,b present the potential map of the lamella region and the eutectic region. It can be seen that there is a definite difference potential between the two phases. The potential of the L12 phase is lower, and the corrosion resistance of the EHEAs is adversely influenced by the galvanic couple effect, which corresponds to the results of EBSD and EDS. In summary, there is a significant difference in the distribution of elements and dislocation between the two phases in the EHEA, resulting in a risk of localized corrosion.

### 3.2. Electrochemical Analysis

**Figure 5:**
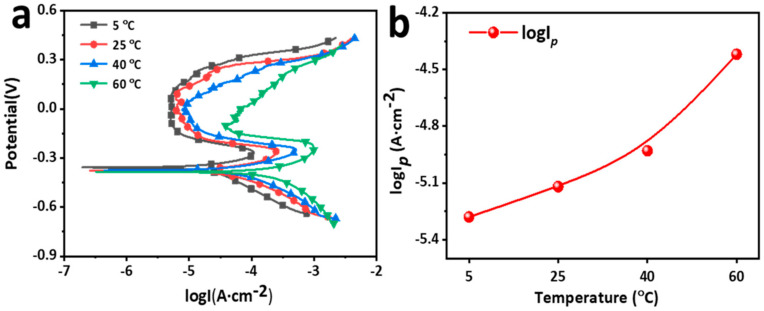
Potentiodynamic polarization curves of AlCoCrFeNi_2.1_ in chloride-containing sulfuric acid solution at different temperatures: (**a**) PDP curve; (**b**) passivity current density.

Figure 5 presents the potentiodynamic polarization curves and passive current density (*i_p_*) of the AlCoCrFeNi_2.1_ EHEA in the sulfuric acid solution containing chloride. The PDP curves reveal typical passivation characteristics. The curves are composed of active, transition, passive and transpassive regions, which is similar to the electrochemical characteristics of Al-containing HEA and 304 stainless steel [35,36]. The polarization curves shift towards the right with an increase in temperature, implying that temperature accelerates both anodic and cathodic reactions on the electrode. The primary passive potential (*E_pp_*) also exhibits no distinct difference at various temperatures, while the primary passive current density (*i_pp_*) increases from 8.82 ×10^−3^ to 9.51 × 10^−2^ A/cm^−2^ as the temperature rises. This indicates that the barrier to passivation increases with temperature. The values of corrosion potentials (*E_corr_*) are nearly −0.38 V at various temperatures, suggesting that temperature does not affect the thermodynamic tendency of corrosion. The passivation transition of the EHEAs at different temperatures is located in the zone ranging from −0.38 V to −0.3 V. The pit potential can be selected when the current density is 0.1 mA/cm^−2^ Kao et al. [18] established that Cl^−^ reacts with Al to generate metastable ion complexes on Al-containing HEA. A temperature rise stimulates the reaction, which is one of the reasons for the poor passive capacity of the EHEA at high temperatures. Overall, *i_p_* raises, transpassive potential (*E_b_*) falls, and the passive range becomes narrower with an increase in environmental temperature, indicating that it is more difficult to passivate the alloy, and the passive film is subject to break at high temperature. The results confirmed that an increase in temperature promotes the dissolution of the EHEAs in chloride-containing sulfuric acid solution containing Cl^−^. In addition, AlCoCrFeNi_2.1_ does not possess satisfactory pitting resistance at relatively high environmental temperatures.

**Figure 6:**
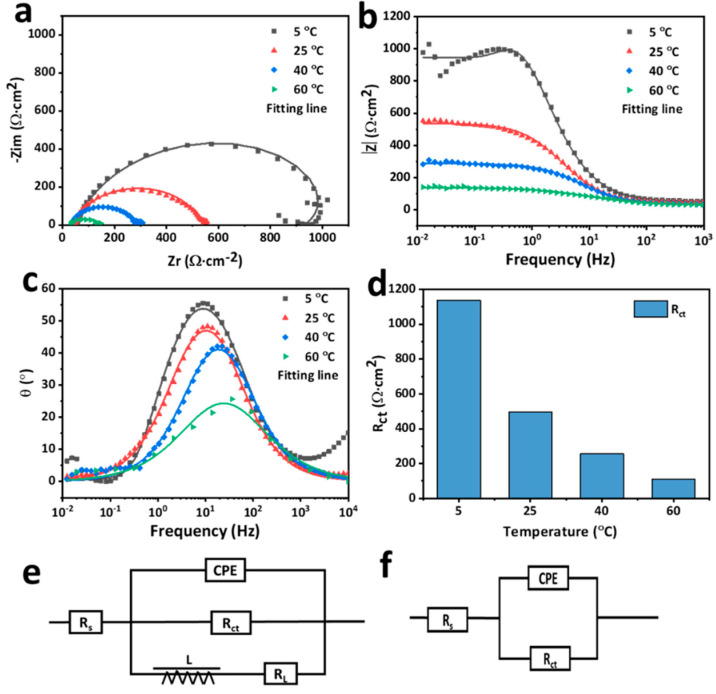
EIS results of AlCoCrFeNi_2.1_ in chloride-containing sulfuric acid solution at different temperatures, (**a**) Nquist map; (**b**) Bode map; (**c**) Phase map; (**d**) Fitting results of *R_ct_*; (**e**) the equivalent electrical circuit at 5 °C; (**f**) the equivalent electrical circuit at 25 °C, 40 °C, and 60 °C.

Electrochemical impedance spectroscopy was employed to study the corrosion behavior of the AlCoCrFeNi_2.1_ EHEA in the sulfuric acid solution at different temperatures. The Nyquist and Bode plots are shown in Figure 6. The diameter of the semi-arc decreases with the increase in temperature. The Nyquist plot of the EHEA at a temperature of 5 °C exhibits a capacitive loop and an inductive loop at high and low frequencies, respectively. However, the Nyquist plot only shows a capacitive loop at 25 °C and above. The capacitive loop is controlled by the charge transfer process, which is related to the interface reaction. Furthermore, the inductive loop is caused by the relaxation and adsorption of H^+^ and Cl^−^ and the dissolution of the oxide layer and the EHEA [37,38]. In Figure 6b, the impedance at 0.1 Hz revealed the polarization resistance in the solutions, and the |impedance with maximum values in the four test temperatures possesses the best corrosion resistance. Figure 6c indicates that the values of the angle fall from 57° to 22° as the temperature from 5 °C to 60 °C. According the Nyquist and Bode plots, the equivalent circuit diagrams in Figure 6e,f are selected to fit the EIS results at 5 °C and other temperatures, respectively. In the graphs, *CPE* represents the constant phase element of double-layer capacitance, *R_s_* is the solution resistance, *R_ct_* denotes the Charge transfer resistance, L represents the inductance, and *R_L_* is the inductance resistor. Due to surface heterogeneity, the capacitance, it is widely accepted that *CPE* can be applied to replace capacitance in the equivalent circuit diagrams. The impedance of *CPE* can be described as follows:

(1) $$Z_{CPE}=\frac{1}{Q{\left(j,\;w\right)}^{n}}$$

where *Q* represents the magnitude of the *CPE*, *j* is an imaginary unit, *w* denotes the angular frequency, and *n* reflects the capacitive character of the *CPE*. The fitting results are presented in Figure 6d and summarized in Table 1. The errors occur during the fitting process and the error values are calculated by software. Temperature slightly affects the value of n at temperatures from 5 ℃ to 40 °C, and the n value at a temperature of 60 °C is marginally lower than at other temperatures, implying that the similar charge transfer process on the EHEA surfaces remains similar at temperatures of 5 °C, 25 °C, and 40 °C. The variable *R_ct_* is used to evaluate corrosion resistance. The value of *R_ct_* decreases sharply with the increase in temperature, since the electrochemical reaction is significantly boosted by the rising temperatures. The results indicate that there is a good correlation between the PDP and EIS data, which suggests that the corrosion resistance of the AlCoCrFeNi2.1 EHEA is unsatisfactory in chloride-containing sulfuric acid solution at higher temperatures.

**Figure 7:**
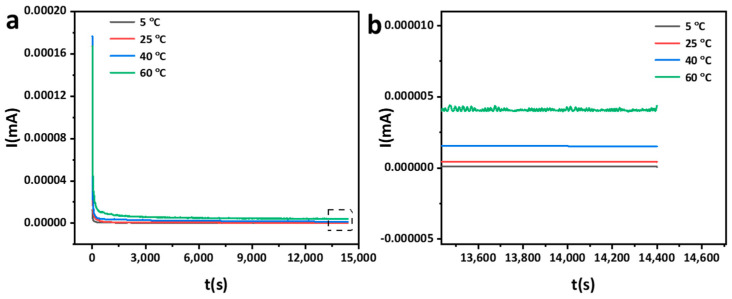
Potentiostatic polarization curves of AlCoCrFeNi_2.1_ in chloride-containing sulfuric acid solution at different temperatures, (**a**) complete image; (**b**) enlarged image.

Potentiostatic polarization is used to characterize the effect of temperature on the passive film of the AlCoCrFeNi_2.1_ EHEA, and the applied potential was −0.1 V_vsSCE_, as Figure 7 shows. Current density drops dramatically in the early stages, which is attributable to the rapid formation of the passive film. Then, it reaches a stable state due to the balance between generation and dissolution of the passive film. Fluctuations in current density in the steady condition increase with temperature, indicating that a leaking passive film with relatively poor protection forms on the EHEA at a higher temperature. The fluctuations of current densities were seen at the end of potentiostatic polarization curves at higher temperatures. Some scholars considered that the fluctuations are related to metastable pitting [39,40].

Mott-Schottky curves are used to study the semiconductor properties and density of the charge carriers in passive films. The Mott-Schottky theory presupposes that the Helmholtz capacitance is much higher than the capacitance of the space charge layer. The space charge capacitance for a semiconductor under depletion conditions can be described by Equations (2) and (3):

(2) $$\frac{1}{C_{SC}^{2}}=\frac{2}{e\varepsilon_{r}\varepsilon_{0}N_{D}}\left(E-\varphi_{fb}-\frac{\kappa T}{e}\right)$$

(3) $$\frac{1}{C_{SC}^{2}}=\frac{2}{e\varepsilon_{r}\varepsilon_{0}N_{A}}\left(E-\varphi_{fb}-\frac{\kappa T}{e}\right)$$

where *C_sc_* is the space charge capacitance, *e* represents the electron charge (1.60218 × 10^−19^ C), *E* denotes the applied potential, *ε_r_* is the dielectric constant of the film, *ε*_0_ is the vacuum permittivity (8.85 × 10^−14^ F/cm), *N_D_* represents the donor density, *N_A_* is acceptor density, *φ_fb_* signifies the flat-band potential, *κ* stands for Boltzmann constant (1.38 × 10^−23^ J/K), and *T* is the absolute temperature.

**Figure 8:**
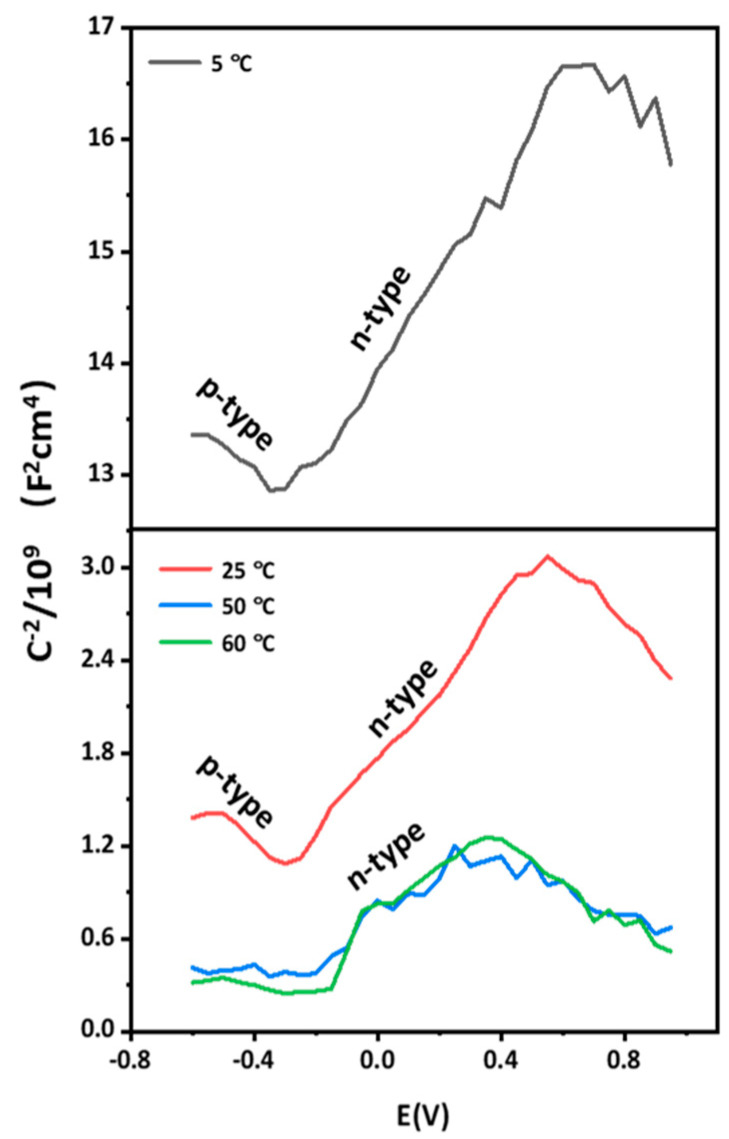
Mott-Schottky curves of the film on AlCoCrFeNi_2.1_ in chloride-containing sulfuric acid solution at different temperatures.

**Figure 9:**
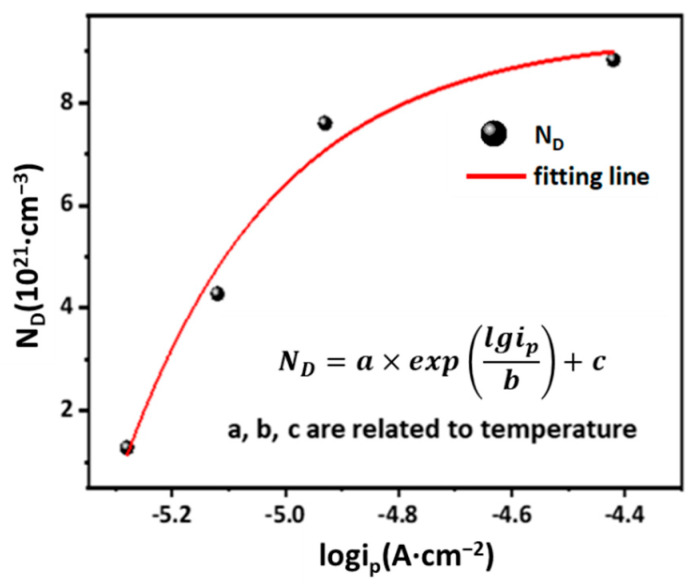
The relationship between *N_D_* and *i_p_* in chloride-containing sulfuric acid solution at different temperatures.

Figure 8 shows the Mott-Schottky curves of the AlCoCrFeNi_2.1_ EHEA after potentiostatic polarization. The curves possess two linear portions with negative and positive slopes at 5 °C and 25 °C, while only positive slopes at 40 °C and 60 °C, implying that the film on the EHEA exhibits the characteristics of a p-n type semiconductor at temperatures of 5 °C and 25 °C and n-type semiconductor at 40 °C and 60 °C. According to conventional wisdom, the outer region of the film, which is composed mainly of Cr oxides, can be considered an n-type semiconductor. In contrast, the inner part of the film consists of Fe oxides and displays p-type semiconductor properties. The negative aspect of the curve shows the electronic feature of a p-type semiconductor, which describes the cation vacancy defect. The linear region with a positive slope reveals that the passive film has the properties of an n-type semiconductor, implying the presence of oxygen vacancies and cation interstitials in the passive film. The semiconductor type is influenced by environmental temperature, which is different from the past reports [26,29]. However, the capacitance value drops noticeably as the temperature increase from 5 °C to 40 °C, while there are minor differences between 40 °C and 60 °C. Changes in hole concentration in the valence are closely related to anion adsorption and result in variations of capacitance value. Fluctuations in capacitance value can be attributed to the electron-depleted layer and charge carriers. Furthermore, the formation of a more disordered passive film at elevated temperatures lead to higher donor density values. The *N_D_* value is obtained from the straight-line portion in the Mott-Schottky curve, as presented in Table 2. Additionally, the variable *φ_fb_* presents similar values at different environmental temperatures. The magnitudes of both *N_D_* and *N_A_* are 10^22^ cm^−3^, which is more than the passive film on stainless steel, implying that the film on the EHEA exhibits poor protection. The value of *N_D_* at 5 °C is the lowest, and *N_D_* decreases slightly as the temperature rises from 25 °C to 60 °C, implying a high solution/metal interface electrochemical reaction at room temperature and above. Passive films with high *N_D_* and *N_A_* values exhibit a certain degree of disorder, and some studies have reported on this influence in the Mott-Schottky curve of metals. Huang et al. [41] stated that the high *N_D_* and *N_A_* values on the passive film of alloy 690 was due to the fast diffusion of metallic ions at high temperature. Cui et al. [29] found that the carrier density of the film on 2057 stainless steel increases with temperature because of the formation rates of oxygen vacancies. According to the point defect model (PDM) devised by Macdonald [42], cation vacancies were generated during the adsorption process of chloride to the active site of oxygen, and a temperature increase enhanced oxygen vacancy formation and anion diffusion. Based on the above findings, although the Fe and Cr content in the film affects the film properties, changes in *N_D_* and *N_A_* not only reveal the defect concentration in the space charge layer, but also demonstrate variation in Fe or Cr oxide. The influence on film composition was analyzed by XPS tests, which revealed that Cl^−^ transmission is controlled by temperature, affecting the value of *N_D_* and *N_A_*. The AlCoCrFeNi2.1 EHEA consists of multiple elements, and the effect of temperature on the electrochemical activity of the different elements varies, which is a critical factor of *N_D_* and *N_A_*. Furthermore, c reflects the ability to repair defects in passive film, and *N_D_* represents the defect density of the passive film. Therefore, there may be an association between *i_p_* and *N_D_*. According to the PDP and M-S results, we have noticed that the *N_D_* shows an exponential relation with log*i_p_* in chloride-containing sulfuric acid solution at different temperatures, as Figure 9 shows.

### 3.3. XPS Analysis

**Figure 10:**
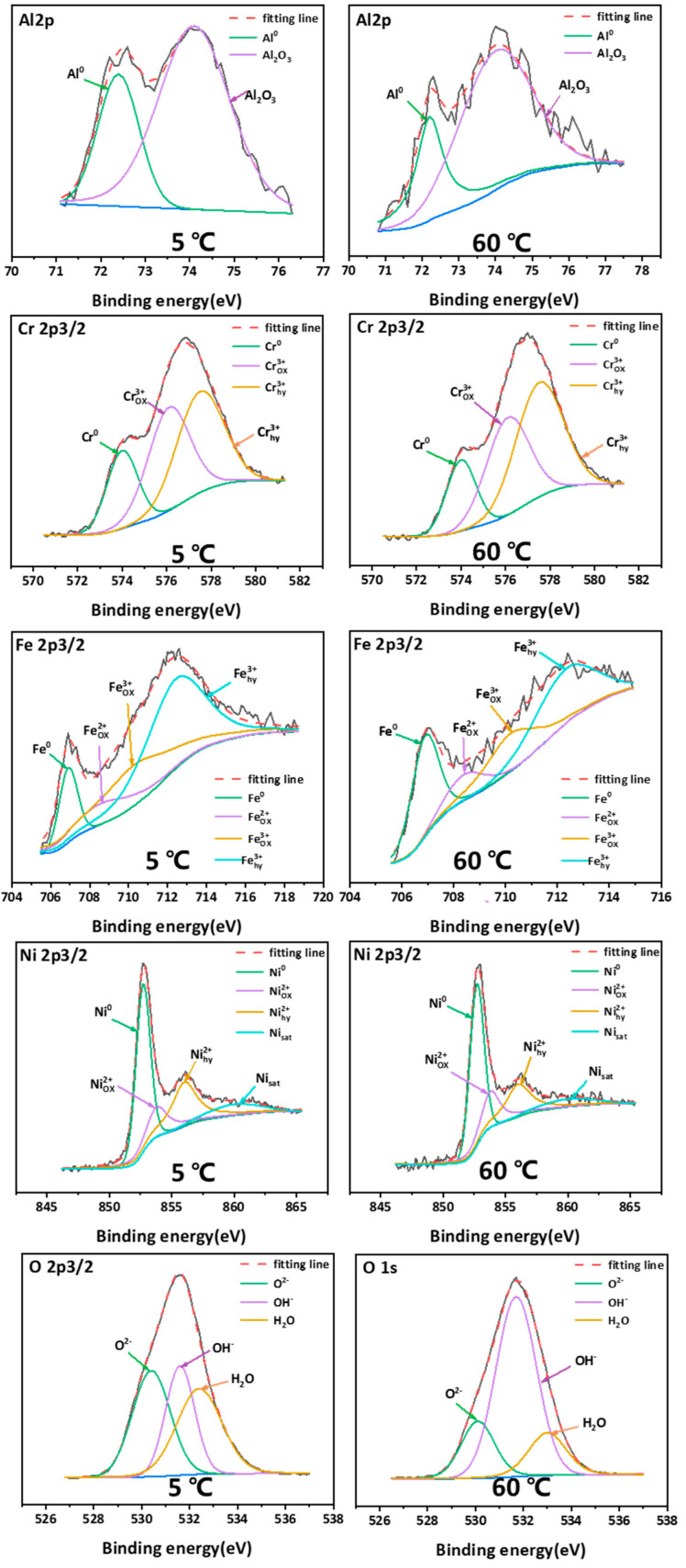
XPS results for Al 2p, Fe 2p3/2, Cr 2p3/2, Ni 2p3/2, and O 1s in the film on AlCoCrFeNi_2.1_ in chloride-containing sulfuric acid solutions at 5 ℃ and 60 ℃.

XPS tests were conducted to investigate the influence of temperature on the film composition. Figure 10 shows the spectra of Al 2p, Cr 2p3/2, Fe 2p3/2, Ni 2p3/2, and O 1s after data processing with the XPSpeak 4.1 software. The Al 2p spectra are separated into two peaks representing metallic Al (72.5 eV) and Al_2_O_3_ (74.7 eV). Due to the slight disparity between the peak of FeO and Fe_2_O_3_, they are not detected separately in previous literature, and many researchers fitted the Fe spectra based on the valence state [12,43]. However, the Fe spectra can be segmented into four distinct peaks representing Fe^2+^ox (708.4 eV), Fe^3^_+ox_ (710 eV), Fe^3+^_hy_ (711.8 eV) and metallic Fe (706.8 eV). Since FeO is unstable, hence the peak of Fe2+ox has been primarily attributed to Fe_3_O_4_. The intensity of the peaks indicates that Fe3+ox and Fe3+hy are the primary forms of Fe in the film on the EHEA at both 5 °C and 60 °C. Cr compounds are considered stable in the passive film on passive metals. Figure 10 shows that Cr^3+^_ox_ (576.2 eV), Cr^3+^_hy_ (557.4 eV), and metallic Cr (573.8 eV) appear in the film at 5 °C and 60 °C. The peak intensities of Cr^3+^_ox_ and Cr^3+^_hy_ are very similar at 5 °C, implying that they are the primary constituents of chromium in the film. Nevertheless, Cr^3+^_hy_ is the main component of the film at 60 °C, owing to its higher peak. This indicates that temperature affects the composition of the film. Cr^3+^_ox_ and Cr^3+^_hy_ are identified as the major components in the passive film in many alloys, and Cr_2_O_3_ contributes to film protection. Combined with the Mott-Schottky curves, we can conclude that the temperature affected the film properties by changing the form of chromium in the film. The Ni 2p3/2 component exhibits four peaks representing Ni^2+^_ox_ (856 eV), Ni^2+^_hy_ (853.9 eV), metallic Ni (852.6 eV), and satellite Ni (859.4 eV), which is used for auxiliary analysis. It has been reported that metallic Ni on the film/matrix interface can hinder ion transmission [44]. In addition, a metallic state and low levels of Ni^2+^_ox_ have been observed in the passive film on stainless steel [29,45]. In this study, due to the high content of Ni in the EHEA, various forms of Ni were detected in the film. The intensity of the three peaks is roughly equal at 5 °C and 60 °C, demonstrating that the form of Ni is not influenced by the temperature. The O 1s are dominated by O^2−^, OH^−^, and H_2_O. O^2−^ and OH^−^ correspond to the formation of Cr, Al, Ni, and Fe hydroxides and oxides. Furthermore, hydroxide is the primary component in the film in chloride-containing sulfuric acid solution.

**Figure 11:**
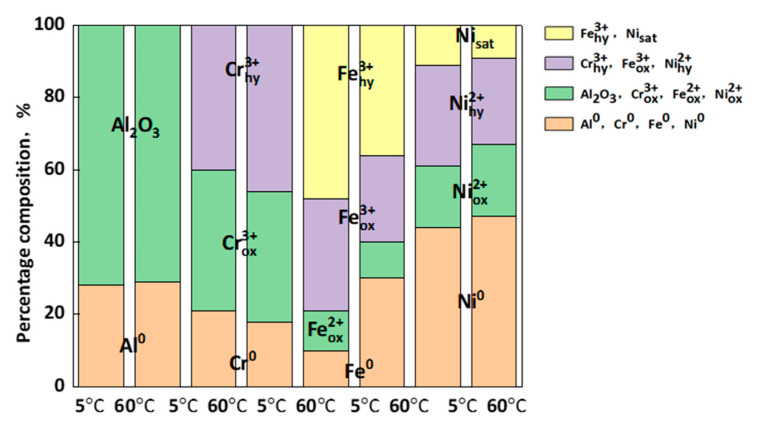
Atomic percentages of the component peaks to the total intensity of Al 2p, Fe 2p3/2, Cr 2p3/2, Ni 2p3/2, and O 1s in the film on AlCoCrFeNi_2.1_ in the acid solutions at 5 ℃ and 60 ℃.

To further study the effect of temperature on the film of the EHEA, semiquantitative analysis of the XPS data was made using fitting software. Figure 11 displays the percentages of Al 2p, Fe 2p3/2, Cr 2p3/2, Ni 2p3/2, and O 1s in the test solutions at 5 ℃ and 60 ℃. Al_2_O_3_ is the main component in the film, and its composition is not affected by temperature. We found that metallic Ni is not the primary form of nickel in the film, while the proportion of oxides and hydroxides is relatively high in the film at tested temperatures. In addition, the ratio of each component was not affected by temperature. An increase in temperature causes a variation in the atomic percentages of the components of Fe 2p3/2 and Cr 2p3/2. Furthermore, the O^2−^/OH^−^ ratio of passive film at 5 ℃ is much higher than at 60 ℃, indicating that a more compact film forms in the acidic solution at low temperature. The temperature has a specific influence on the O^2−^/OH^−^ ratios of chromium and iron but does not change the composition of aluminum and nickel. This suggests that the formations of chromium, iron oxide, and iron hydroxide are the primary factors that influence corrosion resistance between low and high temperatures.

### 3.4. Corrosion Observation

**Figure 12:**
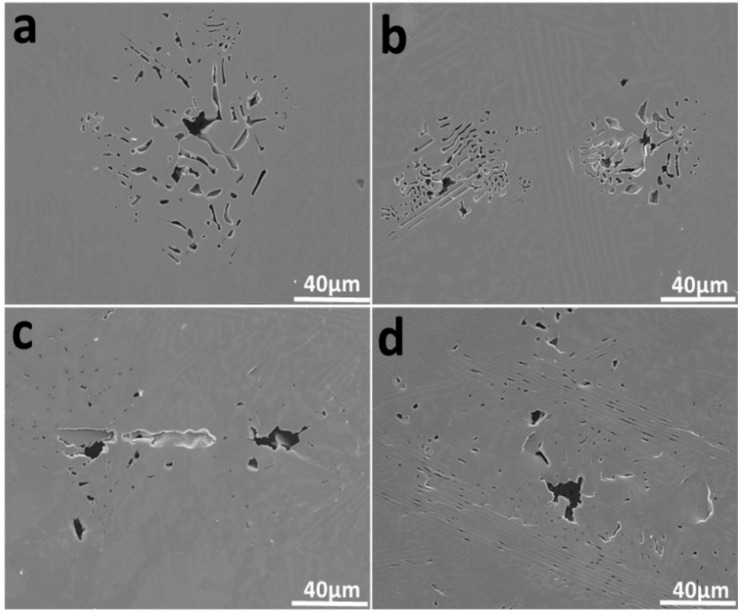
Pitting of AlCoCrFeNi_2.1_ in chloride-containing sulfuric acid solution at: (**a**) 5 ℃; (**b**) 25 ℃; (**c**) 40 ℃; (**d**) 60 ℃.

**Figure 13:**
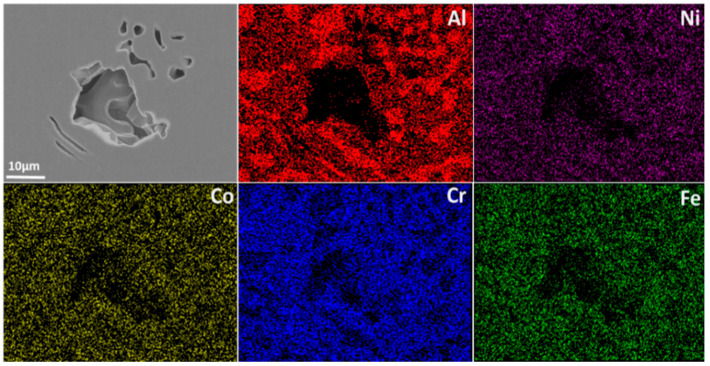
EDS of local corrosion on AlCoCrFeNi_2.1_ in chloride-containing sulfuric acid solution at 5 ℃.

**Figure 14:**
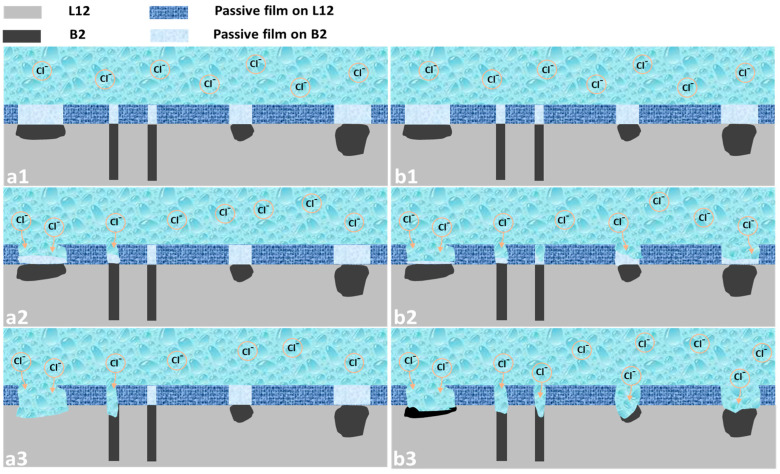
Schematic diagram of corrosion on AlCoCrFeNi_2.1_ in chloride-containing sulfuric acid solution at: (**a1**–**a3**) low temperature; (**b1**–**b3**) high temperature.

The corrosion morphologies of the EHEA at various temperatures after potentiostatic polarization for 30 min were observed using SEM, and the results are shown in Figure 12. In general, the temperature has an obvious influence on the corrosion behavior of the EHEA. Corrosion of the EHEA occurs in both regular and irregular patterns and exhibits apparent selectivity. Only the pitting appears at 5 ℃, while pitting and few selective dissolution regions are observed at 25 ℃. Furthermore, pitting and more selective dissolution zones occur at 40 ℃ and 60 ℃, indicating that an increase in temperature increases the rate of selective dissolution. The distribution of elements around local corrosion was investigated by EDS, as Figure 13 displays. There is almost no aluminum in the pit, a small amount of Ni, and high content of Fe, Cr, and Co. The Al-Ni-rich phases are selectively dissolved. The galvanic effect between the B2 and L12 phase resulted in selective anodic dissolution. This is caused by the heterogeneous distribution of elements and dislocation density (Figure 2 and Figure 3), which is supported by the SKPFM data (Figure 4). It has been previously reported that Al reacts with sulfate radical and hydroxide to generate the chelate compound [18]. The results implied that the Cr-rich phase exhibits better corrosion resistance. Furthermore, the passive film on the Al-Ni-rich phase have a higher Al_2_O_3_ content than L12 phase, resulting in more defect. Thus, the inside of the B2 phase cannot be identified. Based on the above conclusions, the corrosion process of the EHEA in the test solution is schematically described in Figure 14. The passive films of few Al-Ni-rich phases are attacked by Cl^−^ at lower temperature (Figure 14a2), and then occurs localized corrosion (Figure 14a3); while the passive films of Al-Ni-rich phases are damaged (Figure 14b2), and more Al-Ni-rich phases are dissolved at higher temperature (Figure 14b3). Metastable pits are preferentially created on the grain boundary or B2 phase. Pitting is enhanced by the effect of polarization. Due to autocatalysis by the caustic ions, the metastable pits gradually grow in the pitting corrosion area or the selective dissolution region. Moreover, the size of the selective dissolution region increases with temperature, and pitting occurs on the surface at various temperatures.

## 4. Conclusions

The effect of temperature on the passive and corrosion behavior of AlCoCrFeNi2.1 EHEA in chloride-containing sulfuric acid solution is studied using electrochemical tests, XPS analysis, and corrosion morphology observation. The main conclusion is as follows:

(1) Based on potentiodynamic polarization curves, the EHEA exhibits an active-passive-transpassive behavior in chloride-containing sulfuric acid solutions at different temperatures. A temperature rise induces a decrease in the transpassive potential and an increase in passive current, and the cathodic reactions are enhanced by the temperature increase.

(2) A change in temperature affect both the semiconductor type and the doping density of the passive film on the EHEA. The passive film on the EHEA consists of hydroxides and oxides of Al, Fe, Cr, and Ni. Temperature variations alter the ratio of the hydroxides and oxides of Fe and Cr but do not have pronounced effect on Al and Ni. The effect of temperature on passive film is determined by variations in the hydroxides and oxides of Fe and Cr, as well as doping density.

(3) Corrosion of the EHEA occurs preferentially in the Al-Ni-rich phase in the chloride-containing sulfuric acid solution at different temperatures. Pitting is the main form of corrosion at low temperatures, while both pitting and selective dissolution occur at a higher temperature. Moreover, rising temperatures promote selective dissolution.

## Figures and Tables

**Table 1:** EIS fitting results of AlCoCrFeNi_2.1_ in the test solution.

Temperature (℃)	*R_s_* (Ω·cm^2^)	*Q_dl_* (Ω^−1^·cm^−2^·s^n^)	*n*	*R_ct_* (Ω·cm^2^)	*R_L_* (H cm^2^)
5	51.58 ± 1.08	(1.43 ± 0.11) × 10^−4^	0.84 ± 0.011	1138 ± 23.9	2195
25	42.78 ± 0.21	(2.15 ± 0.04) × 10^−4^	0.84 ± 0.004	495.9 ± 3.07	-
40	32.86 ± 0.23	(2.27 ± 0.12) × 10^−4^	0.83 ± 0.006	255.6 ± 1.85	-
60	29.42 ± 0.35	(6.76 ± 0.39) × 10^−4^	0.68 ± 0.012	110.1 ± 1.47	-

**Table 2:** *N_A_* and *N_D_* of AlCoCrFeNi_2.1_ in test solution at different temperatures.

Temperature (°C)	*N_D_* (10^21^·cm^−3^*)*	*N_A_* (10^22^·cm^−3^)
5	1.2788	7.3417
25	4.2757	7.3643
40	7.6035	-
60	8.8372	-

## Data Availability

The raw/processed data required to reproduce these findings cannot be shared at this time as the data is related to an ongoing study.
